# Effect of Core-shell Ceria/Poly(Vinylpyrrolidone) (PVP) Nanoparticles Incorporated in Polymer Films and Their Optical Properties (2): Increasing the Refractive Index

**DOI:** 10.3390/ma10070710

**Published:** 2017-06-27

**Authors:** Toshio Itoh, Toshio Uchida, Noriya Izu, Woosuck Shin

**Affiliations:** National Institute of Advanced Industrial Science and Technology (AIST), Shimo-shidami, Moriyama-ku, Nagoya 463-8560, Japan; to-uchida@aist.go.jp (T.U.); n-izu@aist.go.jp (N.I.); w.shin@aist.go.jp (W.S.)

**Keywords:** core-shell nanoparticles, cerium oxide, poly(vinylpyrrolidone), refractive index, dipentaerythritol hexaacrylate

## Abstract

We investigated the preparation of well-dispersed core-shell ceria-poly(vinylpyrrolidone) (PVP) nanoparticles with an average particle size of around 20 nm which were used to produce a hybrid film with a polymer coating of dipentaerythritol hexaacrylate (DPHA). We obtained good dispersion of the nanoparticles in a mixed solvent of 48% 1-methoxy-2-propanol (MP), 32% 3-methoxy-3-methyl-1-butanol (MMB), and 20% methyl *i*-butyl ketone (MIBK). An ink of the polymer coating consisting of 68.7 wt% nanoparticles and 31.3 wt% DPHA with a polymerization initiator was prepared using this solvent mixture. The surface of the hybrid film showed low roughness and the nanoparticles formed a densely packed structure in the DPHA matrix. The resulting coating possessed excellent transparency and a high refractive index of 1.69.

## 1. Introduction

Polymer coatings have attracted much attention for applications in optical devices, such as lenses and displays. For anti-reflective coating applications, coatings with irregular surfaces for scattering reflected light were investigated [1,2], where control of the refractive index of the coating decreased the intensity of reflected light [3,4,5]. Such coatings should possess a low refractive index, ideally with a value equal to the square root of the refractive index of the substrate [3], however, it is difficult to prepare lower refractive index coatings. In order to solve this problem, a method for producing multilayer coatings having low and high refractive indices has been investigated. The development of transparent films with a high refractive index is required for this structure.

Many research groups have presented methods for improving the properties of polymer coatings, such as modification of the organic materials used in the coatings to increase the refractive index [6,7]; and adding inorganic fillers into the polymer matrix to achieve UV-blocking properties [8,9], improve the mechanical and thermomechanical properties [10,11,12], improve antistatic performance [13], add a hydrophobic property on the surface [14], and increase the refractive index [15,16,17]. For industrial application of these technologies, such investigations should be carried out under the conditions used in industry. Considering the solvent for inks to prepare polymer coatings, methyl *i*-butyl ketone (MIBK) is widely used in industry due to advantages related to volatility, viscosity, and price. It is expected that coatings made from inks containing MIBK and/or other solvents possessing properties corresponding to those of MIBK could be easily applied in industry. Moreover, for the application of such coatings for flat panel displays, it is necessary to investigate the coatability of the ink on polyethylene terephthalate (PET) and triacetylcellulose (TAC) substrates. Therefore, we propose that investigating the development of high-refractive-index transparent coatings should also be carried out under industrially-relevant conditions.

We previously investigated the preparation of UV-blocking hybrid films containing core-shell ceria-poly(vinylpyrrolidone) (PVP) nanoparticles [9]. Cerium oxide possesses high ultraviolet absorbance [18] and a high refractive index [19,20]. Nanoparticles of cerium oxide have been prepared by several different synthesis methods [21,22,23,24,25,26]. We also previously reported the synthesis and characterization of core-shell ceria-PVP nanoparticles [23,24,25,27]. The core-shell ceria-PVP nanoparticles were obtained using the simple polyol method, which was only a reflux of polyethylene glycol solution with cerium nitrate and PVP [23,24,25]. We previously investigated the use of nanoparticles with an average grain size of 37 nm and pentaerythritol triacrylate as a monomer for hard coating materials for UV-blocking coatings. These nanoparticles are difficult to disperse in aprotic solvents, so 20% 3-methoxy-3-methyl-1-butanol (MMB) was mixed with MIBK to improve the dispersibility; we then prepared inks for preparing hybrid films containing 29.0 wt% nanoparticles and 71.0 wt% polymer matrix [9].

**Figure 1:**
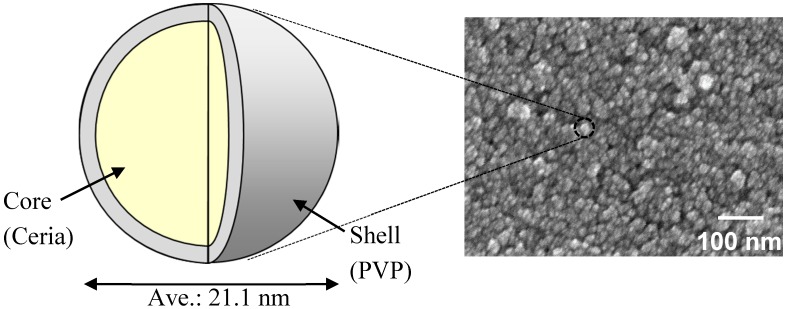
Schematic diagram of core-shell ceria-PVP nanoparticles and a corresponding FE-SEM image showing the nanoparticles with 21.1 nm average particle size.

In this study, we investigated the preparation of hybrid films containing nanoparticles in order to produce a high-refractive-index material. The loading of the nanoparticles in hybrid films should be sufficiently high to increase the refractive index. In this study we increased the loading of the nanoparticles to 68.7 wt%. Since we have empirical knowledge that the polymer matrix of dipentaerythritol hexaacrylate (DPHA) is harder than pentaerythritol triacrylate, the DPHA was used as a monomer in the polymer matrix to maintain the hardness of the film with a high loading. We used nanoparticles with an average particle size of 21.1 nm. Figure 1 shows a schematic diagram of the core-shell ceria-PVP nanoparticles and a corresponding field emission scanning electron microscopy (FE-SEM) image. These particles were sufficiently small to maintain translucency of the hybrid films. In industry, such nanoparticles are supplied as slurries using 1-methoxy-2-propanol (MP) or water as the solvent. The nanoparticles did not disperse well in a solvent mixture of 20% MP/80% MIBK or 20% MMB/80% MIBK, unlike nanoparticles with an average grain size of 37 nm. Firstly, we investigated the preparation of well-dispersed nanoparticle dispersions using a solvent mixture including MIBK and subsequently prepared hybrid films to evaluate their optical properties.

## 2. Materials and Methods

### 2.1. Dispersibility of Core-Shell Ceria-PVP Nanoparticles in Various Solvents

Commercially available water-based slurries of core-shell ceria-PVP nanoparticles with an average particle size of 21.1 nm were provided by Hokko Chemical Industry (Tokyo, Japan), who synthesized the nanoparticles in accordance with our previous report [24]. The average particle size was confirmed by FE-SEM observations, using a JSM-6335FM microscope (JEOL; Tokyo, Japan). The water-based slurry was centrifuged at 18,000 rpm for 90 min to collect the nanoparticles using a Himac CR20GII (Hitachi; Tokyo, Japan) centrifugal separator. Candidate protic solvents for the coating inks are listed in Table 1, along with their viscosities, boiling points, and saturated vapor pressures. We mixed 0.1 g of the nanoparticle sediment with 2 mL of solvent (loading to a nanoparticle concentration around 25 g/L). The mixtures were sonicated at 600 W for 5 min using a UH-600S ultrasonic homogenizer (SMT; Tokyo, Japan). Next, the dispersions were left to stand for five days to test the stability. The state of the dispersion was classified by their dispersion behavior (no dispersion, white cloudy dispersion, yellowish cloudy dispersion, deep yellowish cloudy dispersion, slightly transparent dispersion, or transparent dispersion) and their precipitation behavior (no precipitation, slight precipitation, large amount of precipitation). The particle size distributions of the dispersions in the solvents were analyzed using an FPAR-1000I dynamic light scattering (DLS) analyzer (Otsuka Electronics; Osaka, Japan).

### 2.2. Synthesis of Core-Shell Ceria-PVP Nanoparticle/DPHA Hybrid Films

**Figure 2:**
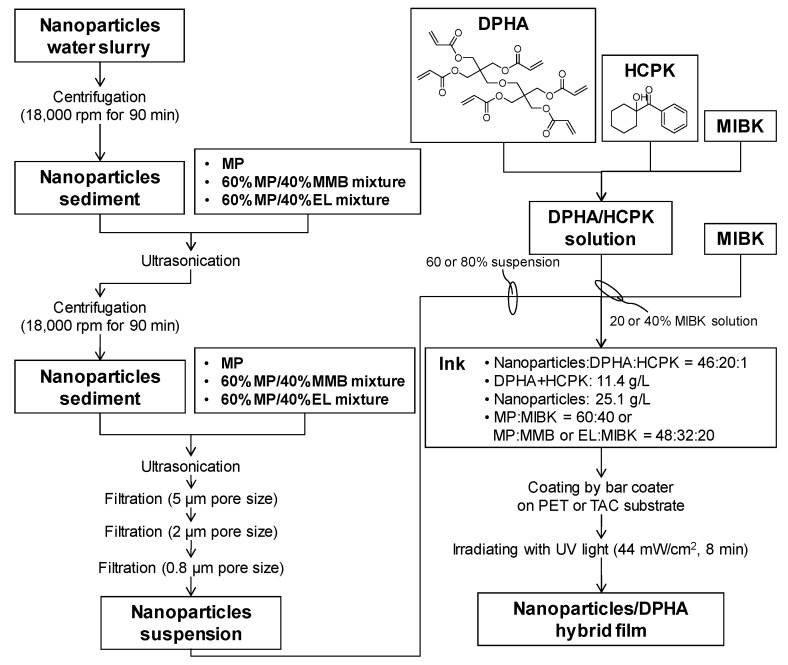
Preparation scheme for the nanoparticle/DPHA hybrid films.

Figure 2 shows the preparation scheme for the nanoparticle/DPHA hybrid films. The water slurry was centrifuged, and the resulting sediment was dispersed in a solvent mixture of MP/EL or MP/MMB by sonication. The centrifuge/sonication process was repeated twice. The suspensions were filtered three times through polypropylene membrane filters with 5, 2, and 0.8 μm pore sizes (Roki Techno; Tokyo, Japan). DPHA as a precursor of the polymer film (57% triester; Shin-Nakamura Chemical, Wakayama, Japan) and 1-hydroxycyclohexyl phenyl ketone (HCPK) as a photopolymerization initiator (Ciba Specialty Chemicals; Basel, Switzerland) were dissolved in MIBK. Inks for making the films were prepared by mixing the nanoparticle suspensions, the DPHA/HCPK solution, and the MIBK. The weight ratio of nanoparticles:DPHA:HCPK was 46:20:1, and the concentrations of the solutes (DPHA and HCPK) and nanoparticles were 11.4 and 25.1 g/L, respectively. The concentration of nanoparticles was 68.7 wt% in the hybrid film when the solvent was evaporated completely. The volume ratios of MP:EL:MIBK and MP:MMB:MIBK were both 48:32:20. The inks were coated on polyethylene terephthalate (PET; Toray; Tokyo, Japan) and triacetylcellulose (TAC; Toray; Tokyo, Japan) substrates, using a K101 bar coater (RK Print Coat Instruments; Litlington, UK). The thickness of the films was controlled by the wire diameter of the bar (#2 with a 124 μm pitch). After coating, the substrates were irradiated for 8 min with UV light using a 1.5 kW mercury lamp equipped with an IR cut-off filter to polymerize the DPHA (Jatec; Tokyo, Japan).

### 2.3. Analysis of Hybrid Films

The surfaces of the hybrid films were observed using FE-SEM. The haze value was measured using an NDH 5000 haze meter (Nippon Denshoku Industries; Tokyo, Japan), equipped with a white light emitting diode as the light source. Refractive indices of the hybrid films were measured using an M-2000 spectroscopic ellipsometry (J. A. Woollmam; Lincoln, NE, USA) over a wavelength range of 195–1680 nm and angles of incidence of 65°, 70°, and 75°. In the calculation of the refractive index, models of the dielectric function and the film thickness were adjusted to minimize the mean square errors of Δ (phase difference) and ψ (amplitude reflectance) between the model and measured data using the WVASE32 software supplied with the instrument.

## 3. Results and Discussion

### 3.1. Dispersibility of Core-Shell Ceria-PVP Nanoparticles in an MP/MIBK Solvent Mixture

As mentioned in introduction, the core-shell ceria-PVP nanoparticles did not disperse sufficiently in the mixtures of 20% MP/80% MIBK and 20% MMB/80% MIBK. As shown in Table 1, the boiling point of MMB is higher than that of MIBK, whereas the boiling point of MP is almost equal to that of MIBK. Considering inks for preparing polymer films, the volatility of the solvents is important for controlling the vaporization rate. Therefore, the use of a high concentration of MMB is not suitable for industrial use because of its low vaporization rate. We firstly investigated increasing the concentration of MP in MP/MIBK mixtures. Table 2 shows the dispersion conditions of the nanoparticles in MP/MIBK and MP solutions. The 40% MP/60% MIBK mixture was able to disperse the nanoparticles, but a cloudy dispersion was obtained. The DLS particle size of the nanoparticles in the 40% MP/60% MIBK solution was 136.5 nm. As the average particle size of the core-shell nanoparticles was 21.1 nm (as shown in Figure 1), it can be concluded that the 40% MP/60% MIBK mixture contained aggregates of the nanoparticles (containing around 100 nanoparticles). When using 100% MP, the DLS particle size of the nanoparticles decreased compared to the case of 40% MP/60% MIBK, indicating a reduction in the degree of aggregation. However, this dispersion was cloudy, indicating insufficient dispersion.

### 3.2. Dispersibility of Core-Shell Ceria-PVP Nanoparticles in Protic Solvents

Subsequently, we investigated other possible solvents; we selected eight protic solvents, including MMB and MP, which possess similar viscosity, boiling point, and saturated vapor pressure values to those of MIBK, MMB, and MP, as shown in Table 1. Table 3 shows the dispersion conditions of the nanoparticles in protic solvents along with their DLS particle sizes. The MMB was able to effectively disperse the nanoparticles, resulting in a slightly transparent dispersion, indicating that the dispersibility of the nanoparticles in MMB was better than in MP. The DLS particle size of the nanoparticles in MMB (69.1 nm) also decreased compared to the case of MP (96.6 nm). Moreover, for ethyl lactate (EL), the resulting dispersion was transparent, and the DLS particle size (56.2 nm) decreased considerably compared to the use of MMB and MP. However, the nanoparticles were not well dispersed in methyl lactate and butyl lactate, where the carbon number of the methylene chain of the ester bond is different from that in EL. Likewise, 3-methoxy-1-butanol, which contains one less methyl group than MMB, was not able to disperse the nanoparticles. In conclusion, the ceria-PVP nanoparticles were not able to be dispersed in aprotic solvents. We also investigated the dispersion of the nanoparticles in several aprotic solvents, in addition to MIBK; no nanoparticle dispersions could be obtained, as shown in the supplementary information (Table S1). This may be due to the protic solvent molecules forming hydrogen bonds with the PVP-shell. In this case, the protic solvent molecules on the surface of the particle were expected to contribute to the dispersibility; however, good dispersion was not obtained in cyclohexane, diacetone alcohol, methyl lactate, butyl lactate, and 3-methoxy-1-butanol, so it is proposed that this is not the only mechanism. The balance between hydrophobicity and hydrophilicity in the molecule may also affect the dispersibility. Therefore, it is difficult to select an appropriate solvent using some parameters from the basic properties of solvents. Hence, in this study, we considered that it is preferable to conduct a dispersibility test in which the solvent ratios of MP, MMB, and EL were adjusted, and subsequently mixed with MIBK.

### 3.3. Relationship between the Condition of Dispersant and the DLS Particle Size

**Figure 3:**
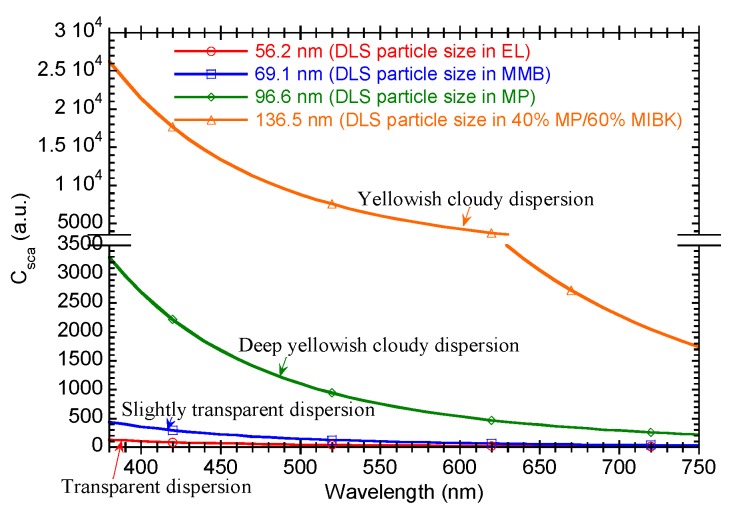
Model of scattering coefficient of dispersions.

When testing various mixing ratios of the MP, MMB, EL, and MIBK solvents, the number of samples can become large, hence, it is necessary to use a method for easily judging the dispersibility. In order to make this analysis more convenient, we evaluated the relationship between the dispersion behavior and the DLS particle sizes in order to be able to characterize the dispersion based only on the dispersion behavior. The conditions of dispersibility can be described by Rayleigh scattering theory [28]. The scattering coefficient, *C_sca_*, is defined by Equation (1):

(1) Csca=83(2πnmD2λ)4((npnm)2−1(npnm)2+2)2·π(D2)2

where *n_m_*, *n_p_*, *λ*, and *D* are the refractive index of the solvent, refractive index of the nanoparticles, wavelength, and diameter of nanoparticles, respectively. Figure 3 shows a model of *C_sca_* calculated using Equation (1) for DLS particle sizes of 56.2, 69.1, 96.6, and 136.5 nm, corresponding to the measured values for the nanoparticles in EL, MMB, MP, and 40% MP/60% MIBK, respectively. Note that the *n_p_* used was that of cerium oxide (2.2) and the *n_m_* of 40% MP/60% MIBK was defined as the weighted average of the two components. The dispersion behavior of 40% MP/60% MIBK dispersion was a yellowish cloudy dispersion; however, it was more white than the dispersion using pure MP as the dispersion behavior of MP dispersion was deep yellowish cloudy dispersion. Figure 3 indicates that the 40% MP/60% MIBK dispersion, with a DLS particle size of 136.5 nm, scattered a large intensity from all regions of the visible spectrum, whereas the scattering intensity from the MP dispersion with a particle size of 96.6 nm, was attenuated, specifically in the longer wavelength region. The dispersion behaviors of the MMB and EL dispersions were slightly transparent dispersion and transparent dispersion, respectively. Figure 3 also indicates that the MMB dispersion showed some scattering behavior at short wavelengths in the visible light region, whereas the EL dispersion showed hardly any scattering. It can be concluded that the dispersion behavior strongly depended on the particle size of the nanoparticles in dispersion. Therefore, for the study of the solvent mixtures, we simply evaluated the dispersion behaviors of the samples, where the DLS particle sizes of some samples were measured for verification.

### 3.4. Dispersibility of Core-Shell Ceria-PVP Nanoparticles in MP, MMB, EL, and MIBK Solvent Mixtures

As mentioned above, we conducted dispersibility tests, in which the ratios of MP, MMB, and EL solvent were adjusted, and then subsequently mixed with MIBK. Among these solvents, MP has a boiling point, viscosity, and saturated vapor pressure the closest to those of MIBK, as shown in Table 1. Since EL and MMB have higher boiling points and lower saturated vapor pressures than MIBK, it is considered that adding large amounts of these solvents would affect the drying process of the film. Therefore, we prepared well-dispersed MP/MMB and MP/EL dispersions at appropriate mixing rates, and subsequently added MIBK.

Table 4 shows the dispersion behaviors of the MP/MMB solvent mixtures. The 40% MP/60% MMB and 60% MP/40% MMB dispersions were slightly transparent, where a small amount of precipitate was observed after five days. Subsequently, MIBK was added to the 40% MP/60% MMB and 60% MP/40% MMB dispersions. Table 5 shows the dispersion behaviors of MP/MMB/MIBK solvent mixtures. The well-dispersed MP/MMB dispersions maintained their slightly transparent state when 20% MIBK was added, while higher concentrations resulted in poorly dispersed (cloudy white) suspensions.

Table 6 shows the dispersion behaviors of MP/EL solvent mixtures. The 40% MP/60% EL and 60% MP/40% EL mixtures resulted in slightly transparent dispersions without any precipitate visible after five days. Subsequently, MIBK was added to the MP/EL dispersions and the dispersion behaviors of the MP/EL/MIBK solvent mixtures are shown in Table 7. The well-dispersed MP/EL dispersions also maintained their slightly transparent condition for MIBK additions up to 20%.

In conclusion, we obtained four kinds of well-dispersed solvent mixtures containing MIBK. As mentioned above, it would be preferable to reduce the amounts of EL and MMB for the film coating process. We therefore used 48% MP/32% MMB/20% MIBK and 48% MP/32% EL/20% MIBK dispersions for preparing films.

### 3.5. Evaluation of Core-Shell Ceria-PVP Nanoparticle/DPHA Hybrid Films

As mentioned in introduction, we prepared hybrid films to evaluate the optical properties using well-dispersed nanoparticle dispersions in this study. The concentration of the nanoparticles in the inks for preparing hybrid films was 25.1 g/L, which was similar to that for the dispersibility test. The volume ratios of MP:EL:MIBK and MP:MMB:MIBK in the inks were both 48:32:20 according to the dispersibility test. The volume ratios of MP:MIBK = 40:60 was also used for the benchmark sample, which was shown to be insufficient dispersed. The weight ratio of nanoparticles:DPHA:HCPK in the inks was 46:20:1 (68.7 wt% of nanoparticles) for the high nanoparticle loading coatings used as high refractive index coatings. 

**Figure 4:**
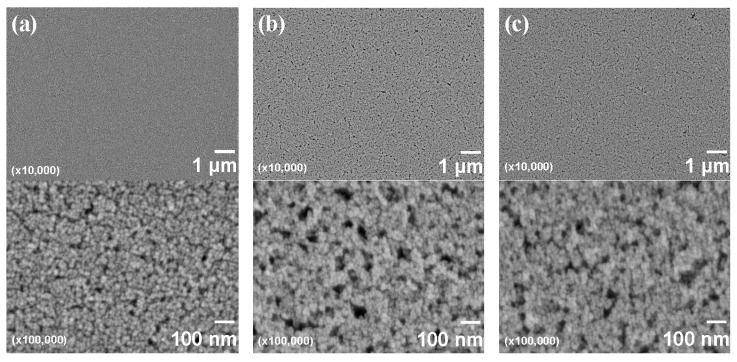
FE-SEM images of nanoparticle-containing DPHA films on PET substrates. The solvents used in the inks were (**a**) 48% MP/32% MMB/20% MIBK; (**b**) 48% MP/32% EL/20% MIBK; and (**c**) 60% MP/40% MIBK.

Figure 4 shows FE-SEM images of nanoparticle-containing DPHA films on PET substrates. The DPHA films were prepared from inks using either 48% MP/32% MMB/20% MIBK, 48% MP/32% EL/20% MIBK, or 60% MP/40% MIBK solvents. Figure 4a shows the low surface roughness of DPHA films from inks using the 48% MP/32% MMB/20% MIBK solvent, while the higher magnification FE-SEM image shows that the nanoparticles were densely packed in the DPHA matrix. In the case of 60% MP/40% MIBK (Figure 4c), the DPHA film had much higher roughness and many pores were observed in the nanoparticle structure. The DPHA film from the 48% MP/32% EL/20% MIBK ink (Figure 4b) also showed significant roughness and many pores, despite the 48% MP/32% EL/20% MIBK showing good dispersion of the nanoparticles.

Therefore, in order to prepare polymer films with densely packed nanoparticle structures, the dispersion condition of the nanoparticles in the inks is important; it is difficult to form densely packed structures from aggregated dispersions. As a result, it is not always possible to prepare DPHA films with a densely-packed nanoparticle structure, even if the solution is well dispersed. We propose that the affinity between the PVP shell of the nanoparticles, DPHA matrix, and solvent is important. This affinity can be explained by the solubility parameter (the indicator of solubility of polymers in solvents). The solubility of the polymer in the solvent is higher when the solubility parameter values of the polymer and the solvent are close to each other [29]. It was reported that the solubility parameters of EL, MP, and MIBK are 20.5, 20.7, and 17.2 (MPa)^1/2^, respectively [29]. The solubility parameters of PVP, DPHA, and MMB were calculated using Equation (2):

(2) $$\delta =\frac{\rho }{M}\sum^{\text{​}}F_{i}$$

where δ, *ρ*, *M*, and *F_i_* denote the solubility parameter, density, molecular weight of the unit, and group molar attraction constants, respectively [29,30]. It has already been reported that the solubility parameters of PVP and MMB are 17.3 and 16.7 (MPa)^1/2^, respectively [9], while that of DPHA is also calculated to be 15.9 (MPa)^1/2^ using group molar attraction constants [29,30,31]. Considering these values, EL and MMB should have low and high affinities for the PVP shell of the nanoparticles and the DPHA matrix, respectively. After coating the inks on the substrates, MP and MIBK with lower boiling points would evaporate easily. Therefore, EL and MMB would affect to the densely-packed nanoparticle structures. Based on the solubility parameter, since EL has low affinity for PVP and DPHA, EL molecules would be localized in DPHA and the nanoparticle matrix. After evaporation of EL, these sites would become pores.

**Figure 5:**
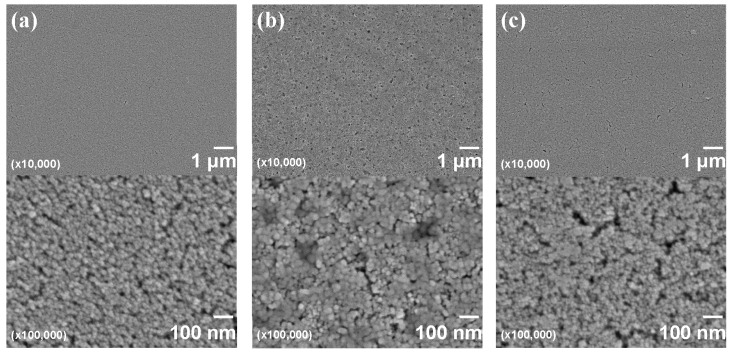
FE-SEM images of nanoparticle-containing DPHA films on TAC substrates. The solvents used in the inks were (**a**) 48% MP/32% MMB/20% MIBK; (**b**) 48% MP/32% EL/20% MIBK; and (**c**) 60% MP/40% MIBK.

Figure 5 shows FE-SEM images of nanoparticle-containing DPHA films on TAC substrates. The characteristics of the films on the TAC substrates using the 48% MP/32% EL/20% MIBK ink was similar to those of the films on the PET substrates, as shown in Figure 5a. The film made from the 60% MP/40% MIBK ink on the TAC substrate also showed more small pores and cracks than that prepared using the 48% MP/32% EL/20% MIBK ink, as shown in Figure 5c. When using the 48% MP/32% EL/20% MIBK solvent mixture, the packed nanoparticle structure was inhomogeneous, as shown in Figure 5b. The TAC substrate was curved when the ink using the 48% MP/32% EL/20% MIBK solvent mixture was coated. Parts of TAC should dissolve into EL, and the dissolving TAC will be educed in homogeneously into the DPHA matrix. Therefore, EL is not appropriate for use with the TAC substrate.

Table 8 shows the refractive indices of nanoparticle-containing DPHA films on PET and TAC substrates, measured at a wavelength of 550 nm. The refractive indices from the films containing many pores and cracks prepared using 60% MP/40% MIBK were different on the PET and TAC substrates. The films with densely packed structures of nanoparticles prepared using 48% MP/32% MMB/20% MIBK ink on PET and TAC substrates showed the highest refractive index (1.69). We observed that the films prepared using poorly dispersed nanoparticles had variable and low refractive indices, while the densely packed structure was not affected by the type of substrate. This indicates that the refractive index is related to the packing structure of the nanoparticles in the DPHA matrix.

**Figure 6:**
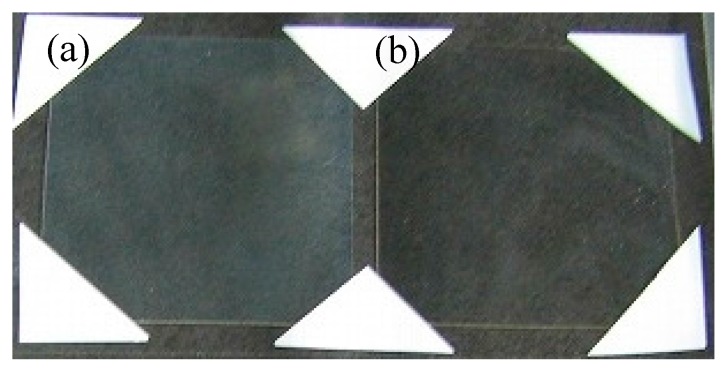
Photographs of nanoparticle-containing DPHA films on PET substrates. The solvents used in the inks were (**a**) 60% MP/40% MIBK and (**b**) 48% MP/32% MMB/20% MIBK.

Figure 6 shows a photograph of nanoparticle-containing DPHA films on PET substrates, prepared using inks with 60% MP/40% MIBK and 48% MP/32% MMB/20% MIBK. In addition, Table 9 summarizes the particle size, thickness, and optical performance of the films shown in Figure 6. The film prepared using the 48% MP/32% MMB/20% MIBK ink (Figure 6b) had higher transparency (in spite of the higher film thickness) than that prepared using 60% MP/40% MIBK ink (Figure 6a). The haze value of the film, *H_film_*, is defined by Equation (3):

(3) $$H_{film}=H_{sample}-H_{substrate}$$

where *H_sample_* and *H_substrate_* are the haze values of the film with the substrate and the substrate without the film (*H_substrate_* of the PET substrate in this study is 1.80), respectively. The haze values also indicate excellent transparency of the film prepared using the 48% MP/32% MMB/20% MIBK ink.

## 4. Conclusions

We investigated the selection of appropriate solvents for the dispersion of core-shell ceria-PVP nanoparticles and the preparation of novel high refractive index hybrid films consisting of DPHA and nanoparticles. In the case of a single solvent, the nanoparticles dispersed well in EL and MMB where the dispersions were slightly transparent. We prepared good dispersions of the nanoparticles in solvent mixtures of 48% MP/32% MMB/20% MIBK and 48% MP/32% EL/20% MIBK. FE-SEM images of the surface of DPHA films prepared from inks containing 48% MP/32% MMB/20% MIBK showed low roughness and a densely packed nanoparticle structure in the DPHA matrix, whereas the DPHA film formed from the 48% MP/32% EL/20% MIBK ink shows high roughness and many pores. We propose that EL had low affinity for the PVP shell and DPHA, so EL molecules would be localized within the DPHA and nanoparticle matrix during evaporation. The resulting coating possessed excellent transparency and a high refractive index of 1.69.

## Figures and Tables

**Table 1:** Protic solvents used in this study.

Name	Structural Formula	Viscosityat 25 °C (cP)	Boiling Point (°C)	Saturated Vapor Pressure at 20 °C (kPa)
Cyclohexanol	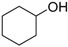	4.6	161	0.13
Diacetone alcohol	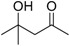	3.05	168	0.21 (26.7 °C)
Methyl lactate	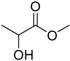	2.88	145	0.36
Ethyl lactate (EL)	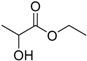	2.58	155	0.36
Butyl lactate	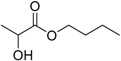	3.35	188	0.05
3-Methoxy-3-methyl-1-butanol (MMB)	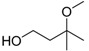	6.12	174	0.5
3-Methoxy-1-butanol	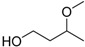	No data	161	No data
1-Methoxy-2-propanol (MP)	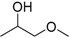	1.78	120	0.89
Methyl *i*-butyl ketone (MIBK)	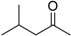	0.62	114-117	2.1

**Table 2:** Dispersion behaviors of the nanoparticles in MP/MIBK and MP solvents.

Ratio of Solvent (vol%)		Dispersion Behavior	DLS (nm)
MP	MIBK		
40	60	Yellowish cloudy dispersion with slight precipitation	136.5
100	0	Deep yellowish cloudy dispersion with slight precipitation	96.6

**Table 3:** Dispersion behaviors of the nanoparticles in protic solvents. The bold text indicates positive results for the next step of mixing with MIBK.

Name	Dispersion Behavior	DLS (nm)
Cyclohexanol	No dispersion	–
Diacetone alcohol	No dispersion	–
Methyl lactate	White cloudy dispersion	–
**Ethyl lactate (EL)**	**Transparent dispersion**	**56.2**
Butyl lactate	White cloudy dispersion	–
**3-Methoxy-3-methyl-1-butanol (MMB)**	**Slightly transparent dispersion with slightly precipitation**	**69.1**
3-Methoxy-1-butanol	White cloudy dispersion	–
**1-Methoxy-2-propanol (MP)**	**Deep yellowish cloudy dispersion with slightly precipitation**	**96.6**

**Table 4:** Dispersion behaviors of the nanoparticles in MP/MMB solvent mixtures. The bold text indicates positive results for the next step of mixing with MIBK.

Ratio of Solvent (vol%)		Dispersion Behavior	DLS (nm)
MP	MMB		
20	80	Yellowish cloudy dispersion with large amount of precipitation	83.5
**40**	**60**	**Slightly transparent dispersion with slight precipitation**	**47.0**
**60**	**40**	**Slightly transparent dispersion with slight precipitation**	**46.9**
80	20	Yellowish cloudy dispersion with large amount of precipitation	75.6

**Table 5:** Dispersion behaviors of the nanoparticles in MP/MMB/MIBK solvent mixtures. The bold text indicates the most positive result for the next step of preparing the nanoparticle/DPHA hybrid films.

Ratio of Solvent (vol%)			Dispersion Behavior	DLS (nm)
MP	MMB	MIBK		
**48**	**32**	**20**	**Slightly transparent dispersion with slight precipitation**	**66.6**
36	24	40	White cloudy dispersion	–
24	16	60	White cloudy dispersion	–
32	48	20	Slightly transparent dispersion with slight precipitation	–
24	36	40	White cloudy dispersion	–
16	24	60	White cloudy dispersion	–

**Table 6:** Dispersion behaviors of the nanoparticles in MP/EL solvent mixtures. The bold text indicates positive results for the next step of mixing with MIBK.

Ratio of Solvent (vol%)		Dispersion Behavior	DLS (nm)
MP	EL		
20	80	Slightly transparent dispersion with slight precipitation	47.8
**40**	**60**	**Slightly transparent dispersion**	**60.3**
**60**	**40**	**Slightly transparent dispersion**	**56.4**
80	20	Yellowish cloudy dispersion with large amount of precipitation	76.2

**Table 7:** Dispersion behaviors of the nanoparticles in MP/EL/MIBK solvent mixtures. The bold text indicate the most positive result for the next step of preparing the nanoparticle/DPHA hybrid films.

Ratio of Solvent (vol%)			Dispersion Behavior	DLS (nm)
MP	EL	MIBK		
**48**	**32**	**20**	**Slightly transparent dispersion with slight precipitation**	–
36	24	40	White cloudy dispersion	–
24	16	60	White cloudy dispersion	–
32	48	20	Slightly transparent dispersion with slight precipitation	–
24	36	40	White cloudy dispersion	–
16	24	60	White cloudy dispersion	–

**Table 8:** Refractive indices of nanoparticle-containing DPHA films on PET and TAC substrates measured at a wavelength of 550 nm.

Solvent of Dispersant in Ink	Refractive Index (λ = 550 nm)	
	PET Substrate	TAC Substrate
48% MP/32% MMB/20% MIBK	1.694	1.689
48% MP/32% EL/20% MIBK	1.656	–
60% MP/40% MIBK	1.636	1.586

**Table 9:** Summary of nanoparticle size, thickness, and optical performance of the films shown in Figure 6.

Item	Figure 6a	Figure 6b
Particle size of the nanoparticles and its coefficient of variation (CV) by FE-SEM	21.1 nm 15.0%	21.1 nm 15.0%
DLS particle size of the nanoparticles in solvents	137.9 nm (in 60% MP/40% MIBK)	46.9 nm (in 48% MP/32% MMB/20% MIBK)
Film thickness	198 nm	325 nm
Refractive index (λ = 550 nm)	1.636	1.694
Haze; *H_film_*	1.98	0.37

## Supplementary Materials

The following are available online at www.mdpi.com/1996-1944/10/7/710/s1, Table S1: Dispersion conditions of the nanoparticles in aprotic solvents.
