# Supplementary material for: Effect of Core-shell Ceria/Poly(Vinylpyrrolidone) (PVP) Nanoparticles Incorporated in Polymer Films and Their Optical Properties (2): Increasing the Refractive Index
Source: Materials (Basel). 2017 Jun 27;10(7):710. doi: 10.3390/ma10070710 (PMC5551753; doi:10.3390/ma10070710)
Supplement: Supplementary file 1 [file materials-10-00710-s001.docx]

Supporting Information

Effect of Core–Shell Ceria/Poly(Vinylpyrrolidone) (PVP) Nanoparticles Incorporated in Polymer Films and Their Optical Properties (2): Increasing the Refractive Index

Toshio Itoh,* Toshio Uchida, Noriya Izu, and Woosuck Shin

National Institute of Advanced Industrial Science and Technology (AIST), Shimo-shidami, Moriyama-ku, Nagoya 463-8560, Japan

**Table S1.** Dispersion behaviors of the nanoparticles in aprotic solvents.

| Name | Structural formula | Viscosity  at 25°C  [cP] | Boiling  point  [°C] | Saturated  vapor pressure  at 20 °C  [kPa] | Condition |
| --- | --- | --- | --- | --- | --- |
| Methyl i-butyl ketone (MIBK) |  | 0.62 | 114-117 | 2.1 | No dispersion |
| 3-Methoxy-3-methyl-1-butylacetate |  | 1.8 | 188 | 0.05 | No dispersion |
| Ethylene glycol monoethyl ether acetate |  | 1.3 | 156 | 0.27 | No dispersion |
| Propylene glycol monomethyl ether acetate |  | 1.2 | 120 | 1.2 | No dispersion |
| Bis(2-ethoxyethyl) ether |  | No data | 180-190 | 0.48 | No dispersion |

© 2017 by the authors. Licensee MDPI, Basel, Switzerland. This article is an open access article distributed under the terms and conditions of the Creative Commons Attribution (CC BY) license (http://creativecommons.org/licenses/by/4.0/).
